# Enhanced cycling stability of NiCo_2_S_4_@NiO core-shell nanowire arrays for all-solid-state asymmetric supercapacitors

**DOI:** 10.1038/srep38620

**Published:** 2016-12-07

**Authors:** Yuanyuan Huang, Tielin Shi, Shulan Jiang, Siyi Cheng, Xiangxu Tao, Yan Zhong, Guanglan Liao, Zirong Tang

**Affiliations:** 1 State Key Laboratory of Digital Manufacturing Equipment and Technology, Huazhong University of Science and Technology, Wuhan 430074, China; 2 Wuhan National Laboratory for Optoelectronics, Huazhong University of Science and Technology, Wuhan 430074, China; 3 Tribology Research Institute, Southwest Jiaotong University, Chengdu 610031, China

## Abstract

As a new class of pseudocapacitive material, metal sulfides possess high electrochemical performance. However, their cycling performance as conventional electrodes is rather poor for practical applications. In this article, we report an original composite electrode based on NiCo_2_S_4_@NiO core-shell nanowire arrays (NWAs) with enhanced cycling stability. This three-dimensional electrode also has a high specific capacitance of 12.2 F cm^−2^ at the current density of 1 mA cm^−2^ and excellent cycling stability (about 89% retention after 10,000 cycles). Moreover, an all-solid-state asymmetric supercapacitor (ASC) device has been assembled with NiCo_2_S_4_@NiO NWAs as the positive electrode and active carbon (AC) as the negative electrode, delivering a high energy density of 30.38 W h kg^−1^ at 0.288 KW kg^−1^ and good cycling stability (about 109% retention after 5000 cycles). The results show that NiCo_2_S_4_@NiO NWAs are promising for high-performance supercapacitors with stable cycling based on the unique core-shell structure and well-designed combinations.

With the increasing demand in energy and environmental protection, the development of high performance energy storage devices has become urgent. Supercapacitors have attracted vast attentions due to the advantages like fast charge-recharge ability, high specific capacity and long cycle life compared with other traditional energy storage devices such as rechargeable fuel cells and batteries^1^,^2^,^3^,^4^,^5^,^6^,^7^,^8^. Supercapacitors are commonly divided into electric double layer capacitors (EDLCs) which store energy using ion adsorption and pseudocapacitors using frequently reversible redox reactions in electrode surface. Pseudocapacitive materials such as metal oxides and electronically conducting polymers have been extensively studied owing to their high theoretical specific capacitance, high energy densities, low cost, and low toxicity compared with most commercial supercapacitor materials^9^,^10^.

Recently, metal sulfides such as Ni_3_S_2_^11^,^12^, CoS^13^ and MoS_2_^14^ have been applied to pseudocapacitors as promising electrode materials owing to their great electrochemical performance like high reversible capacity and good electrical conductivity synergistically. Among them, NiCo_2_S_4_ is outstanding owing to its higher reversible capacity, richer redox reactions and more sensitive electrical conductivity than the other metal sulfides^15^,^16^. NiCo_2_S_4_ has been widely studied for supercapacitor applications in the past few years. For example, NiCo_2_S_4_ nanosheets grown on reduced graphene oxide (RGO) present a high specific capacitance of 1161 F g^−1^ at the current density of 5 A g^−1^ (4.5% loss after 2,000 cycles)^15^. NiCo_2_S_4_ nanosheets grown on Nitrogen-doped carbon foams show a great specific capacitance of 8.77 F g^−1^ at the current density of 20 A g^−1^ (9.6% loss after 2,000 cycles)^17^. NiCo_2_S_4_ nanotubes grown on Ni foam exhibit a specific capacitance of 738 F g^−1^ at the current density of 4 A g^−1^ (6.6% loss after 4,000 cycles)^18^. NiCo_2_S_4_ porous nanotubes through a sacrificial template method show a specific capacitance of 1093 F g^−1^ at a current density of 0.2 A g^−1^ (15.5% loss after 5,000 cycles)^19^. However, bare NiCo_2_S_4_ electrode often results in poor cycleability and low energy density because of the occurrence of redox reactions, the insufficient contact between the active material and electrolyte, and the instable structure during the electrochemical reaction. In this regard, well-designed NiCo_2_S_4_-based hybrid nano-architectures with other well-known metal oxides/hydroxide capacitive materials may be a good way to meet the requirement of high-performance supercapacitors^20^,^21^,^22^,^23^,^24^. In the meantime, NiO is widely studied for supercapacitors as the positive electrode material due to its high theoretical specific capacitance of 2573 F g^−1^ within 0.5 V^25^, good electrochemical stability^26^, practical availability, environmentally benign nature and low cost.

Herein, we developed a facile and low-cost process to fabricate an original three-dimensional core-shell structure on Ni foam with NiCo_2_S_4_ nanowires and NiO nanosheets as core and shell, respectively. NiCo_2_S_4_ nanowires synthesized through two-step hydrothermal reactions acted as skeleton supporting for the NiO shell. NiO nanosheets were coated on the surface of NiCo_2_S_4_ nanowires by electrochemical deposition and post-annealing subsequently. The core-shell structure can provide abundant redox reaction sites, facilitate the sufficient contact of electrode and electrolyte, and enhance the cycleability. The new electrode demonstrates a remarkable specific capacitance (12.2 F cm^−2^ at the current density of 1 mA cm^−2^) and enhanced cycling performance (the capacity retention of 89% over 10,000 cycles). To further evaluate the NiCo_2_S_4_@NiO NWAs electrode for practical applications, an all-solid state ASC was fabricated. The assembled device receives a superior energy density of 30.38 W h kg^−1^ at 0.288 KW kg^−1^, outstanding power density of 0.72 KW kg^−1^ at 10.36 W h kg^−1^ and good cycling stability (109% retention after 5,000 cycles). The results demonstrate that NiCo_2_S_4_@NiO NWAs are the kind of promising electrode with enhanced cycling stability for high performance supercapacitor applications. The methodology through well-designed combinations and fabrication method presented in this work are applicable for the development of the energy storage devices with a wide variety of excellent capacitive materials.

## Results and Discussion

**Figure 1:**
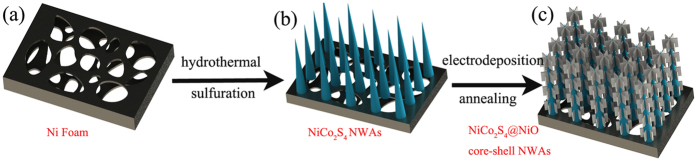
Schematic illustrating the formation process of the NiCo_2_S_4_@NiO NWAs on Ni foam. (**a**) Ni foam, (**b**) NiCo_2_S_4_ NWAs, (**c**) NiCo_2_S_4_@NiO NWAs.

The electrode fabrication procedure of NiCo_2_S_4_@NiO NWAs is schematically shown in Fig. 1. Firstly, NiCo_2_S_4_ nanowires were densely grown on Ni foam through a hydrothermal and sulfuration process. Later, the NiCo_2_S_4_ nanowires were acted as a scaffold for the growth of NiO nanosheets through electrochemical deposition and post-annealing process. The NiO nanosheets can act as an armor to protect the integrity of NiCo_2_S_4_ nanowires surviving from reversible redox reactions.

**Figure 2:**
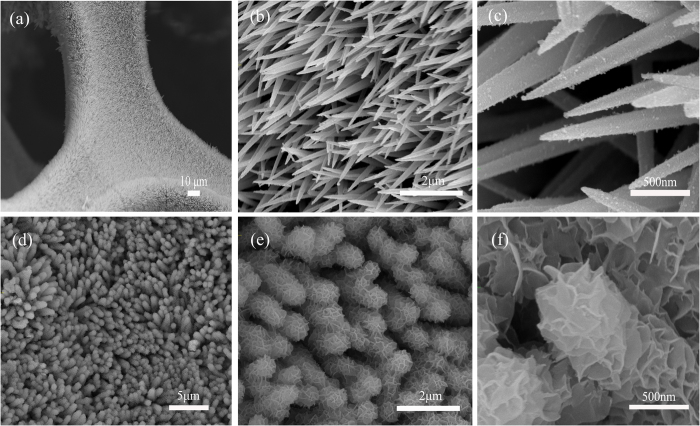
SEM images of NiCo_2_S_4_ NWAs (**a,b** and **c**) at different magnifications; SEM images of NiCo_2_S_4_@NiO NWAs (**d,e**, and **f**) at different magnifications.

A series of characterizations were carried out to study the morphologies of the NiCo_2_S_4_@NiO NWAs. Figure 2 presents typical SEM images of the NiCo_2_S_4_ nanowires and NiCo_2_S_4_@NiO NWAs supported on the 3D porous Ni Foam substrate. From Fig. 2(a), Ni foam is completely covered by orderly NiCo_2_S_4_ nanowires. Enlarged SEM images of Fig. 2(b), 2(c) reveal that the surface of NiCo_2_S_4_ nanowires is relatively smooth. After electrodeposited for 10 minutes and annealed, the NiCo_2_S_4_ nanowires are covered by thin NiO nanosheets, as shown in Fig. 2(d), forming a core-shell hierarchical nanostructure. From the enlarged images of Fig. 2(e) and (f), the thin NiO nanosheets are connected with each other, forming the unique core-shell structure. The obtained core-shell structure greatly enlarges the surface area, offering abundant redox reactions sites.

**Figure 3:**
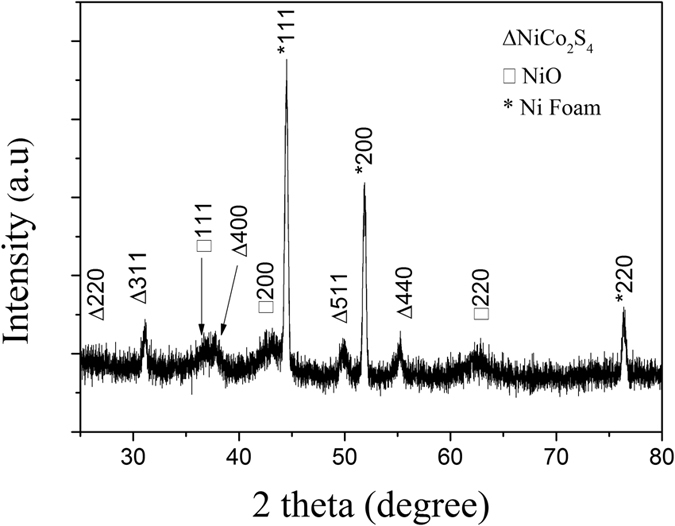
XRD pattern of the hierarchical NiCo_2_S_4_@NiO core-shell nanowires scratched from NiO foam.

The Fig. 3 shows the corresponding X-ray diffraction (XRD) patterns of the as-synthesized sample. The three typical peaks at 44.7°, 52.1° and 76.5° are respectively identified as (111), (200) and (220) planes of the Ni foam. The four major peaks at 31.5°, 38.3°, 50.4° and 55.3° can be respectively identified as (311), (400), (511) and (440) planes of the NiCo_2_S_4_ phase (JCPDS card No. 20–0782)^27^,^28^,^29^,^30^. Moreover, the diffraction peaks at 37.2°, 43.3°, 62.9° coincide with (111) (200) (220) plane respectively in the standard NiO spectrum (JCPDS card No. 47-1049). And no additional diffraction peak is detected, which confirms that the hybrid structure consists of NiCo_2_S_4_ and NiO only.

**Figure 4:**
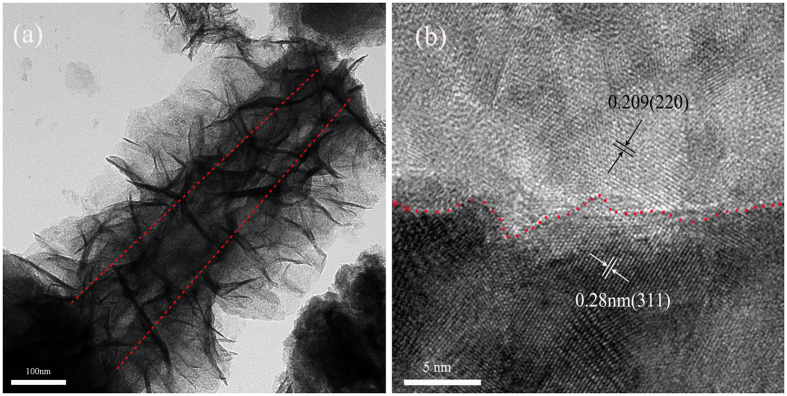
(**a**) TEM image of the NiCo_2_S_4_@NiO core-shell nanowire. (**b**) HRTEM image of the NiCo_2_S_4_@NiO core-shell nanowire.

The detailed morphology and core-shell structure of NiCo_2_S_4_@NiO were confirmed by TEM, high-resolution TEM (HRTEM), as shown in Fig. 4 and EDX mapping in Figure S1. The core-shell structure of NiCo_2_S_4_@NiO is clearly shown in Fig. 4(a), where the core and the shell can be distinguished by the illustrated red lines. The HRTEM image further verifies the core-shell nanostructure, as exhibited in Fig. 4(b). The heterointerface between the NiCo_2_S_4_ core and the NiO shell can be obviously distinguished by the red line. Top part shown in Fig. 4(b) reveals the inner NiO nanocrystallite, which has a lattice fringe of 0.209 nm, assigning to the (200) crystal plane of NiO. Bottom part in Fig. 4(b) reveals the lattice fringe of 0.28 nm which is close to the (311) crystal plane of NiCo_2_S_4_. These results well agreed with XRD patterns. The EDX mapping of the NiCo_2_S_4_@NiO NWAs is also displayed in Figure S1, which further confirm the multiple core-shell structure of the composite.

Electrochemical performance of the NiCo_2_S_4_@NiO NWAs

**Figure 5:**
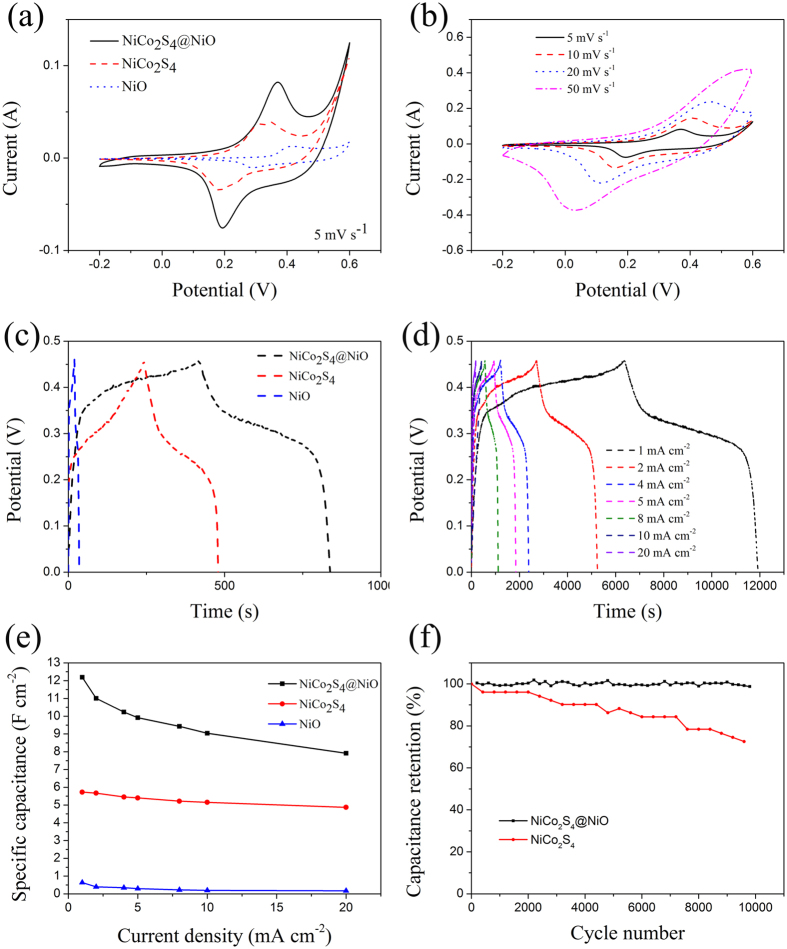
(**a**) Comparison of CV curves of NiCo_2_S_4_@NiO core-shell NWAs, NiCo_2_S_4_ NWAs and NiO nanosheets at the scan rate of 5 mV s^−1^. (**b**) The CV curves of NiCo_2_S_4_@NiO core-shell NWAs electrode at different scan rates. (**c**) Comparison of GCD curves of NiCo_2_S_4_@NiO core-shell NWAs, NiCo_2_S_4_ NWAs and NiO nanosheets at the current density of 10 mV cm^-2^. (**d**) The GCD curves of NiCo_2_S_4_@NiO core-shell NWAs electrode at different current densities. (**e**) Geometric specific capacitances of NiCo_2_S_4_@NiO core-shell NWAs, NiCo_2_S_4_ NWAs and NiO nanosheets electrodes at different current densities. (f) Cycling properties of NiCo_2_S_4_@NiO core-shell NWAs electrode and NiCo_2_S_4_ NWAs electrode at the current density of 20 mA cm^−2^ for 10,000 cycles.

Electrochemical measurements were carried out in a three-electrode electrochemical cell with 3 M KOH as the electrolyte. Figure 5(a) shows respective CV curves over a potenial range from ^−^0.2 to 0.6 V for the NiCo_2_S_4_@NiO, NiCo_2_S_4_ and NiO electrode at 5 mV s^−1^. Obviously, at the same scan rate, the integrated area of the NiCo_2_S_4_@NiO NWAs is much larger than that of the NiCo_2_S_4_ or NiO nanostructure electrodes, which indicates that the NiCo_2_S_4_@NiO NWAs electrode has the highest capacity. As shown in Fig. 5b, with the increment of scan rate, the voltammetric current of the NiCo_2_S_4_@NiO NWAs electrode increases. The redox peaks in each CV curve demonstrate the pseudocapacitive properties of the NiCo_2_S_4_ and NiO, which may owe to Ni^2+^/Ni^3+^, Co^2+^/Co^3+^ and Co^3+^/Co^4+^ transitions as shown in the following equations^17^,^26^.


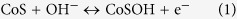



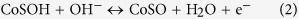



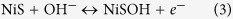



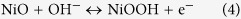


Galvanostatic charge-discharge (GCD) tests were also carried out to estimate the electrochemical performance of the NiCo_2_S_4_@NiO NWAs electrode. Figure 5(c) shows the comparison of the charge and discharge curves of the NiCo_2_S_4_@NiO NWAs, NiCo_2_S_4_ NWAs and NiO nanosheets, respectively. The NiCo_2_S_4_@NiO NWAs electrode reveals vastly longer charge-discharge time than both NiCo_2_S_4_ nanowires and NiO nanosheets at the same current density. And Fig. 5(d) shows the GCD curves of NiCo_2_S_4_@NiO NWAs electrode at various current densities varying from 1 to 20 mA cm^−2^. According to the equation (7) and (8), the specific capacitance was calculated with the discharge time and the corresponding results were plotted in Fig. 5(e). Evidently, the NiCo_2_S_4_@NiO delivers a high specific capacitance 12.2 F cm^−2^ at the current density of 1 mA cm^−2^. And the calculated results of the specific capacitance based on the active mass was also provided in Figure S2. Obviously, the NiCo_2_S_4_@NiO NWAs electrode delivers much higher areal specific capacitance than the NiCo_2_S_4_ nanowire arrays or NiO nanosheets, and also higher than many previously reported electrodes based on NiCo_2_S_4_, such as NiCo_2_S_4_@Ni_(1−x)_Co_x_(OH)_2_ core-shell nanoarrays (3.54 F cm^−2^ at 1 mA cm^−2^)^31^, NiCo_2_S_4_ nanotube@Ni-Mn Layered Double Hydroxide arrays/graphene sponge (1.74 F cm^−2^ at 1 mA cm^−2^)^28^, NiCo_2_S_4_ nanotube@ NiCo_2_S_4_ nanosheet arrays on Ni foam (4.38 F cm^−2^ at 5 mA cm^−2^)^32^ and NiCo_2_S_4_@MnO_2_ heterostructures (2.6 F cm^−2^ at 3 mA cm^−2^)^33^. The high specific capacitance of the electrode can be attributed to the specific core-shell arrays structure and the well-designed combination of NiCo_2_S_4_ with NiO. The 3D core-shell structure also enables easy access of electrolyte, and promotes the transport of electrolyte and the contact between the electrode and electrolyte. And the NiCo_2_S_4_ nanowire arrays electrode possesses not only good pseudocapacitive behavior itself, but also provides vast electron passageways. The thin NiO nanosheets can enhance the surface area and protect the NiCo_2_S_4_ nanowire surviving from redox reactions.

The measurement of cycling performance for the NiCo_2_S_4_@NiO NWAs and NiCo_2_S_4_ NWAs electrodes were shown in Fig. 5(f). The overall loss of NiCo_2_S_4_@NiO NWAs after 10,000 cycles was less than 11.15%, much better than the NiCo_2_S_4_ electrode’s 29.42%. Obviously, the cycling stability has tremendously improved after the compositing. Comparing with NiCo_2_S_4_ nanowire arrays, the enhanced stability of NiCo_2_S_4_@NiO core-shell arrays can be attributed to the coaxial structure and the well-designed combination, in which the NiCo_2_S_4_ core offers a solid skeleton to interlink the NiO nanosheets, and the NiO layer protects the NiCo_2_S_4_ structural from destroyed in electrolyte. As a result, the mutual cooperation ensures the structural integrity of NiCo_2_S_4_@NiO nanocomposites and thus the enhanced cycling stability. And to the best of our knowledge, the cycling stability of NiCo_2_S_4_@NiO NWAs is also much higher than previously reported NiCo_2_S_4_ based electrodes, such as NiCo_2_S_4_ nanosheets grown on Nitrogen-doped carbon foam (9.6% capacity loss after 2,000 cycles)^17^, NiCo_2_S_4_ nanotube@Ni^−^Mn (11.7% capacity loss after 1,000 cycles)^28^, CoxNi_1-X_ (OH)_2_/NiCo_2_S_4_ nanotube (4% capacity loss after 2,000 cycles)^34^.

**Figure 6:**
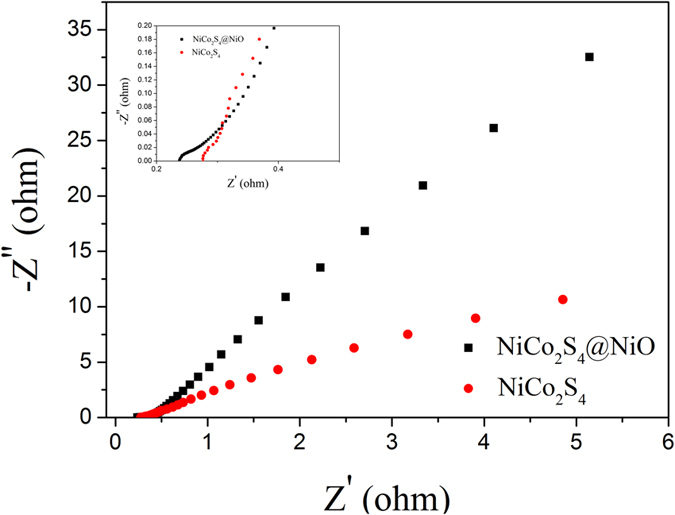
Nyquist plots of NiCo_2_S_4_@NiO and NiCo_2_S_4_.

Electrochemical impedance spectroscopy (EIS) tests had been further conducted to observe the intrinsic mechanism on the dramatic performance improvement of the NiCo_2_S_4_@NiO nanocomposites. Figure 6 depicts the Nyquist plots of the EIS spectra for the NiCo_2_S_4_@NiO and NiCo_2_S_4_ electrode, respectively. The charge transfer resistance R_ct_ of the hybrid NiCo_2_S_4_@NiO NWAs electrode (0.03 Ω) is lower than that of the pristine NiCo_2_S_4_ NWAs electrode (0.09 Ω), which was deduced by the dimeter of the semicircle^35^. NiCo_2_S_4_@NiO shows lower internal resistance Re (0.237 Ω) than NiCo_2_S_4_ (0.275 Ω). This result clearly reveals that the NiCo_2_S_4_@NiO demonstrates better charge-transfer kinetics and quick ion transport than NiCo_2_S_4_.

Comparing with NiCo_2_S_4_ nanowire arrays, the smart design of NiCo_2_S_4_@NiO NWAs shows outstanding electrochemical performance, which can be included the following advantages. (i) Both NiCo_2_S_4_ and NiO have high specific capacitance, and exhibit excellent capacitive behavior in the same KOH alkaline electrolyte, thereby contributing to the increase of overall capacitance significantly. (ii) The NiCo_2_S_4_ core has an intimate electrical connection to the NiO nanosheets, which builds a reliable conductive network for quick ion transport. The thin NiO nanosheets can enlarge the effective contact surface area, which enables the full exposure of the active materials to the electrolyte. The core-shell structure could provide short pathways for the ion diffusion and rapid charge collection/transfer, and the enlarged surface area make more sufficient contact between the electrolyte and the electrode^17^,^29^,^30^. Therefore, the maximum harvest of pseudocapacitance can be achieved from the NiCo_2_S_4_@NiO core-shell nanostructure^36^,^37^,^38^,^39^. (iii) The combination of NiCo_2_S_4_ and NiO with different redox reaction potential results in more electro active sites for the Faradaic redox reactions^40^,^41^,^42^. (iv) Comparing with the bare NiCo_2_S_4_ NAWs, the three-dimensional core-shell structure of the NiCo_2_S_4_@NiO NAWs can retard the inside strain which caused by the volume changes during the cycling, The NiCo_2_S_4_ NWAs are served as rigid back-bones to support NiO by interlinking the polymeric chains, the NiO nanosheets coated on the NiCo_2_S_4_ NWs may relieve the stress exerted on inner nanowires caused by severe volume change, and thus suppress the degradation of the NiCo_2_S_4_ core^43^,^44^,^45^. Thus, the *in-situ* combination of NiCo_2_S_4_ and NiO would give rise to a strong synergetic effect and good mechanical integrity for improving the electrochemical performance and cycling stability. In summary, the composed electrode of NiCo_2_S_4_@NiO NWAs can achieve vastly enhanced performance than bare NiCo_2_S_4_.

Electrochemical performance of the NiCo_2_S_4_@NiO//AC all-solid-state ASCs

**Figure 7:**
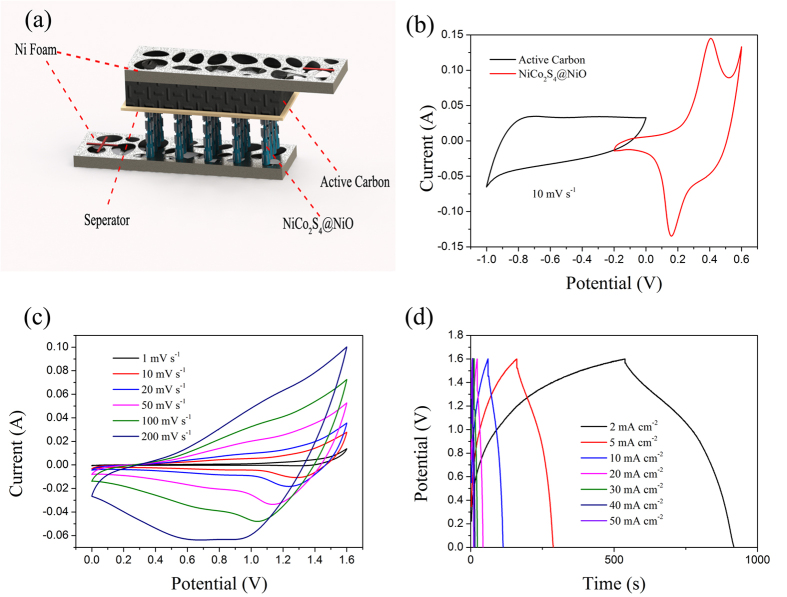
(**a**) Schematic illustration of an ASC that composed of the positive electrode of NiCo_2_S_4_@NiO NWAs, separator, and the negative electrode of AC; (**b**) CV curves of NiCo_2_S_4_@NiO NWAs and AC half cells in 3 M KOH solution at the scan rate of 10 mV s^−1^; (**c**) CV curves of the ASC tested at different scan rates ranging from 1 to 200 mV s^−1^; (**d**) Galvanostatic charge-discharge curves at different current densities from 2 to 50 mA cm^−2^

To further evaluate the NiCo_2_S_4_@NiO NWAs electrode for practical applications, the ASC was fabricated using the NiCo_2_S_4_@NiO NWAs as the positive material and the AC film as the negative material, as shown in Fig. 7(a). To investigate the potential range of the device, the CV curves collected in a three-electrode system from the AC film electrode and NiCo_2_S_4_@NiO NWAs hybrid electrode in 3 M KOH electrolyte were demonstrated in Fig. 7(b). The CV curve of the AC is a nearly rectangular shape without redox peaks, which shows classic EDLCs behavior. And the CV curves of NiCo_2_S_4_@NiO electrode shows two pairs of redox peaks in the potential window from ^−^0.2 to 0.6 V, attributing to the typical pseudocapacitance character^46^,^47^. In order to obtain the optimal performance of the ASC device, the charge between the positive and the negative electrodes should be balanced following the relationship q^+^ = q^−^. The charge stored by each electrode depends on the specific capacitance (C), the potential range for the charge/discharge process (ΔE) and the mass of the electrode (m) following the equation (5)^48^:


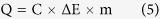


And the mass ratio will follow the equation (6):


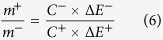


According the equation, the mass ratio between the NiCo_2_S_4_@NiO electrode and AC electrode was calculated to be around 1:1.8. To investigate the influence of scan rate on the electrochemical performance, the rate-dependent CV curves of the NiCo_2_S_4_@NiO//AC ASC device with scan rates from 10 to 200 mV s^−1^ were measured in Fig. 7(c). Notably, at the high scan rate of 200 mV s^−1^ and the maximum working voltage of 1.6 V, the shape of the CV curve is still well retained, denoting a good rate capability.

**Figure 8:**
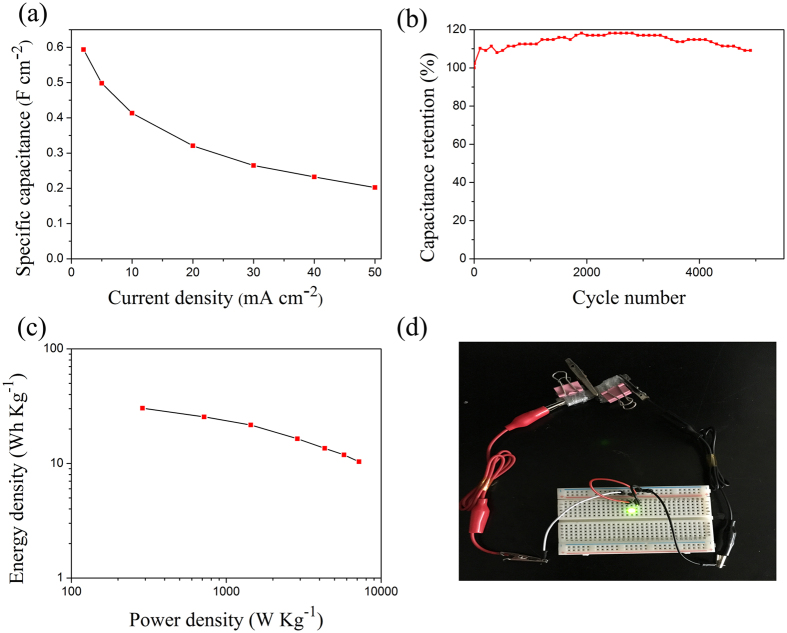
(**a**) Specific capacitance of the ASC at different current densities; (**b**) Cycling performance of ASC devices collected at the scan rate of 20 mA cm^−2^; (**c**) Ragone plots of energy density and power density of NiCo_2_S_4_@NiO//AC; (**d**) Optical images showing that two NiCo_2_S_4_@NiO//AC all-solid-state ASCs in series lighting up a green LED indicator.

Galvanostatic charge-discharge measurements were conducted between 0 to 1.6 V to calculate the specific capacitance of NiCo_2_S_4_@NiO//AC. Figure 7(d) shows the galvanostatic charge-discharge curves for the different current densities from 2 mA cm^−2^ to 50 mA cm^−2^ in the potential window of 0 ^−^ 1.6 V. During the charge-discharge, the charge curves of the NiCo_2_S_4_@NiO//AC ASCs are almost symmetric to its corresponding discharge counterpart, even at the high current density of 200 mA cm^−2^. The capacitance of NiCo_2_S_4_@NiO//AC device with different current densities was calculated according to equation (7) and (8), which was plotted in Fig. 8(a). The capacitance of NiCo_2_S_4_@NiO//AC changed from 0.59 to 0.21 F cm^−2^ with the augment of the current density from 2 to 50 mA cm^−2^. As shown in Fig. 8(b), the capacitance was slowly increased during the first cycle because only a fraction of material was active. And after more cycles of the charge and discharge, the NiCo_2_S_4_@NiO core-shell nanowires became fully activated and contributed to the large increase of the capacitance. The specific capacitance of the hybrid electrode still retained about 109% of its initial value after 5,000 cycles. According to equation (9) and (10), the energy density and power density were calculated and shown in Fig. 8(c).The maximum energy density of the ASC was further calculated to be 30.385 W h Kg^−1^ at the power density of 0.288 KW Kg^−1^. At the high discharge current of 20 mA cm^−2^, the energy density still remained10.36 W h Kg^−1^ at the power density of 0.72 KW Kg^−1^. The highest energy density obtained here is superior than that of many reported ASC devices based on NiCo_2_S_4_, such as the NiCo_2_S_4_ nanosheet//AC with the energy density of 25.5 W h Kg^−1^ 49, the NiCo_2_S_4_//AC with the energy density of 22.8 W h kg^−1^ at 0.16 W kg^−1^ 50, the NiCo_2_S_4_ (nanosheets)//AC with the energy density of 22.4 W h kg^−1^ at 0.335 kW kg^−1^ 49 and mesoporous NiCo_2_S_4_ (nanoparticles)//AC with the energy density of 28.3 W h kg^−1^ at 0.245 kW kg^−1^ 51. Compared with a sequence of other ASC devices based the core-shell structure, like CNT@NiO//PCPs (25.4 W h kg^−1^ at 0.4 kW kg^−1^) 52, Ni(OH)@3DNi//AC (21.8 W h kg^−1^ at 0.66 kW kg^−1^) 46 and NiCo_2_O_4_@NiMoO_4_//AC (21.7 W h kg^−1^ at 0.157 kW kg^−1^) 41, the result of NiCo_2_S_4_@NiO//AC device developed here also possesses a competitive superiority. In order to further demonstrate the potential application of the ASC, a green light-emitting diode (LED) indicator was powered after charging to 3.2 V for 20 s, as shown in Fig. 8(d). These results show outstanding performance of the ASCs and prove that the as-obtained NiCo_2_S_4_@NiO NWAs are promising for practical applications.

In general, we have exhibited a low-cost hydrothermal synthesis with a subsequent electrochemical deposition process for the fabrication of the three-dimensional NiCo_2_S_4_@NiO NWAs on Ni foam. The NiCo_2_S_4_@NiO NWAs electrode exhibited superior performance with high specific capacitance of 12.2 F cm^−2^ at the current density of 1 mA cm^−2^, a remarkable cycling stability (about 89% retention after 10,000 cycles). The fabricated all-solid-state ASC device based on the NiCo_2_S_4_@NiO core-shell electrode also demonstrated excellent electrochemical performance in terms of energy density (30.385 W h Kg^−1^ at 0.288 k W Kg^−1^), cycling lifespan (109% retention after 5000 cycles). The ASC was further assembled in series to verify their practical application, for LED indicator as the example. We anticipate that this work will advance the development of supercapacitors with metal sulfide materials, and the methodology through unique structural and well-designed combinations are also applicable to improve the electrochemical performance for energy-storage devices.

## Methods

### Synthesis of NiCo_2_S_4_ nanowire arrays

All the chemicals used in this study were purchased without further purification. In a typical procedure, the Ni foam was cleaned by 5% HCl, ethanol and deionized (DI) water to remove impurities and oxides. Then, 1.903 g of CoCl_2_⋅6H_2_O, 0.95076 g of NiCl_2_, and 0.72072 g of urea were dissolved in 60 mL DI water. Later, transferring the solution into a 100 ml Teflon-lined autoclave and putting a piece of clean Ni foam in the autoclave. Then the autoclave was put in the oven and the oven was kept at 120 °C for 6 h. After cooling down to room temperature, the precursors were received after washing with DI water and ethanol for several times. Next, 4.8 g sodium sulfide was dissolved in 60 ml DI water. Then the solution was transferred into the 100 ml Teflon-lined autoclave together with the as-obtained precursors, and the autoclave was kept in the oven under 160 °C for 6 h. Finally, the NiCo_2_S_4_ nanowire arrays were obtained through washing the sample with DI water and ethanol for several times, and then dried at 60 °C for 12 h. The mass loading of NiCo_2_S_4_ was around 9.1 mg cm^−2^.

Synthesis of NiCo_2_S_4_@NiO core-shell nanowire arrays

Ni(OH)_2_ nanosheets were synthesized through an electrochemical deposition process. The as-fabricated NiCo_2_S_4_ nanowires on Ni foam acted as the working electrode, a saturated SCE acted as the reference electrode and a Pt sheet acted as the counter electrode. The electrochemical deposition process was conducted in the solution of 0.1 M NiNO_3_ at the potential of ^−^1 V. Different electrochemical deposition duration of 1, 5, 10, 15, and 20 minutes was respectively selected to find the optimal loading of NiO for the composite electrode. Then, the obtained samples were washed by DI water, dried at 60 °C for 12 h. Finally, the NiCo_2_S_4_@NiO NWAs supported on Ni foam were synthesized by annealing the as-obtained NiCo_2_S_4_@NiO NWAs on Ni foam in air atmosphere at 300 °C for 2 h. The CV curves of the electrodes were showed in Figure S3 and more discussions were provided in the support information. Based on the CV curve analysis, we chose 10 minutes of electrochemical deposition as the optimal deposition duration. In the manuscript, all of the NiCo_2_S_4_@NiO NWAs related tests were based on it. The mass loading of NiCo_2_S_4_@NiO NWAs with electrochemical deposition of 10 minutes was around 13.48 mg cm^−2^.

Synthesis of NiO nanosheets arrays

First of all, the Ni foam was cleaned by 5% HCl, ethanol and deionized (DI) water to remove impurities and oxides. Using the same method mentioned previously, Ni(OH)_2_ nanosheets were synthesized via the electrochemical depositing process. Ni foam acted as the working electrode, a saturated SCE acted as the reference electrode and a Pt sheet acted as the counter electrode. The electrochemical deposition was conducted in the solution of 0.1 M NiNO_3_ at the potential of ^−^1 V for 10 minutes. Then, the obtained sample was washed by DI water, dried at 60 °C for 12 h. Finally, the NiO nanosheets supported on Ni foam were synthesized by annealing the as-obtained NiOH nanosheets on Ni foam in air atmosphere at 300 °C for 2 h. The mass loading of NiO was around 1 mg cm^−2^.

### Characterization

The morphologies of the samples were characterized by scanning electron microscopy (SEM, FEI Nova NanoSEM 450) and transmission electron microscopy (TEM, FEI Tecnai G2 S-TWIN). The crystallographic phases of the NiCo_2_S_4_@NiO NWAs were characterized by X-ray diffraction (XRD) with radiation from a Cu target (Kα, λ = 0.154 nm).

### Electrochemical measurements

The electrochemical performance of as-prepared NiCo_2_S_4_ NWAs, NiCo_2_S_4_@NiO NWAs and NiO nanosheets samples were all evaluated using an electrochemical Autolab workstation (PGSTAT-302N, Eco Echemie B.V. Company). The cyclic voltammetry (CV) and electrochemical impedance spectroscopy (EIS) measurements were taken by the three-electrode cell in 3 M KOH aqueous electrolytes. Galvanostatic charging/discharging (GCD) and cycling tests of the electrodes were conducted using a battery measurement system (LAND CT2001A). The EIS measurements were conducted with a frequency range from 10^−2 ^Hz to 10^5^ Hz and voltage amplitude of 10 mV at open-circuit potential. The areal specific capacitance (C_a_) and mass specific capacitance (C_m_), energy density (E), and power density (P) were calculated by following equations^47^:


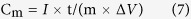



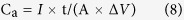



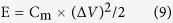



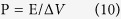


where *I, t*, m, Δ*V*, and A respectively respresents the discharge current (mA), the discharge time (s), the total mass of active materials (g), the potential window of the electrode (V), and the surface area of the electrode (cm^2^).

Fabrication of all-solid-state asymmetric supercapacitor devices

The ASCs were assembled using NiCo_2_S_4_@NiO NWAs as the positive electrode and activated carbon slurry by mixing 80 wt% AC, 10 wt% carbon black with 10 wt% polytetrafluorene-ethylene (PTFE) as the negative electrode and a piece of filter paper as the separator. The PVA-KOH gel electrolyte was obtained by mixing 3 g polyvinyl alcohol (PVA), 1.63 g KOH with 30 ml of DI water.

## Additional Information

**How to cite this article**: Huang, Y. *et al*. Enhanced cycling stability of NiCo_2_S_4_@NiO core-shell nanowire arrays for all-solid-state asymmetric supercapacitors. *Sci. Rep.* **6**, 38620; doi: 10.1038/srep38620 (2016).

**Publisher's note:** Springer Nature remains neutral with regard to jurisdictional claims in published maps and institutional affiliations.

## Supplementary Material

Supplementary Information
