# Supplementary material for: Enhanced cycling stability of NiCo2S4@NiO core-shell nanowire arrays for all-solid-state asymmetric supercapacitors
Source: Sci Rep. 2016 Dec 7;6:38620. doi: 10.1038/srep38620 (PMC5141571; doi:10.1038/srep38620)
Supplement: Supplementary Information [file srep38620-s1.pdf]

Supplementary information

Enhanced cycling stability of $NiCo_2S_4$@NiO core-shell nanowire arrays for all-solid-state asymmetric supercapacitors

Yuanyuan Huang[1], Tielin Shi[1,2], Shulan Jiang[3], Siyi Cheng[1], Xiangxu Tao[1], Yan Zhong[1], Guanglan Liao[1,2], and Zirong Tang[1,2*]

[1] State Key Laboratory of Digital Manufacturing Equipment and Technology, Huazhong University of Science and Technology, Wuhan 430074, China

[2] Wuhan National Laboratory for Optoelectronics, Huazhong University of Science and Technology, Wuhan 430074, China

[3] Tribology Research Institute, Southwest Jiaotong University, Chengdu 610031, China

*corresponding author, 1037 Luoyu Road, Wuhan 430074, China. Tel.: +86 27 87792241; fax: +86 27 87792413; E-mail address: zirong@hust.edu.cn (Z. Tang)

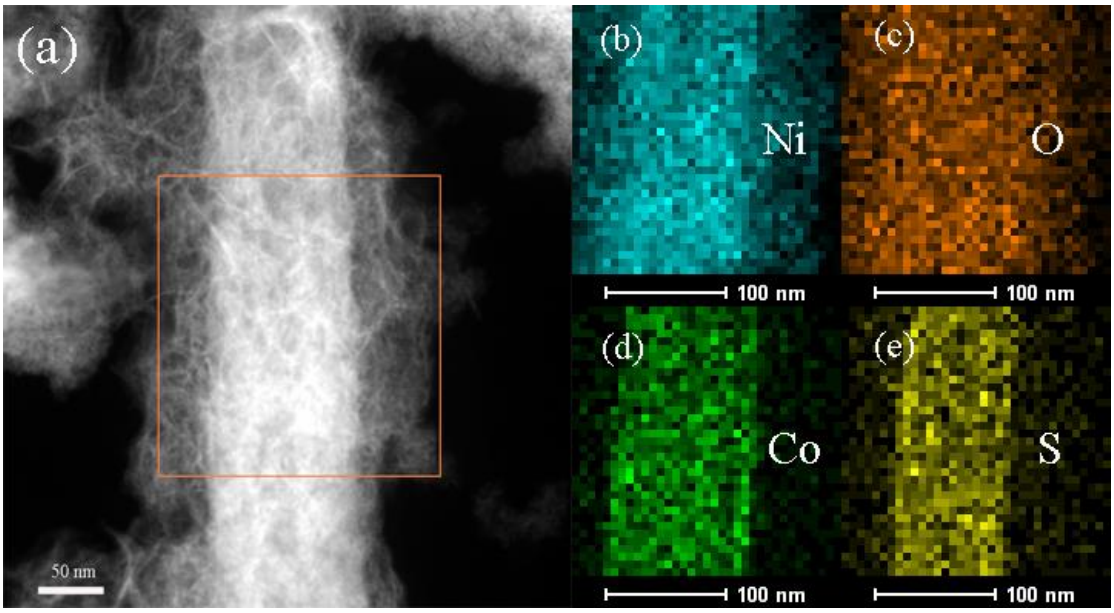

Figure S1. (a) High Angle Annular Dark Field (HAADF) image of the $NiCo_2S_4$@NiO NWAs and its corresponding electron energy loss spectroscopy elemental mapping of (b) Ni, (c) O, (d) Co, (e) S.

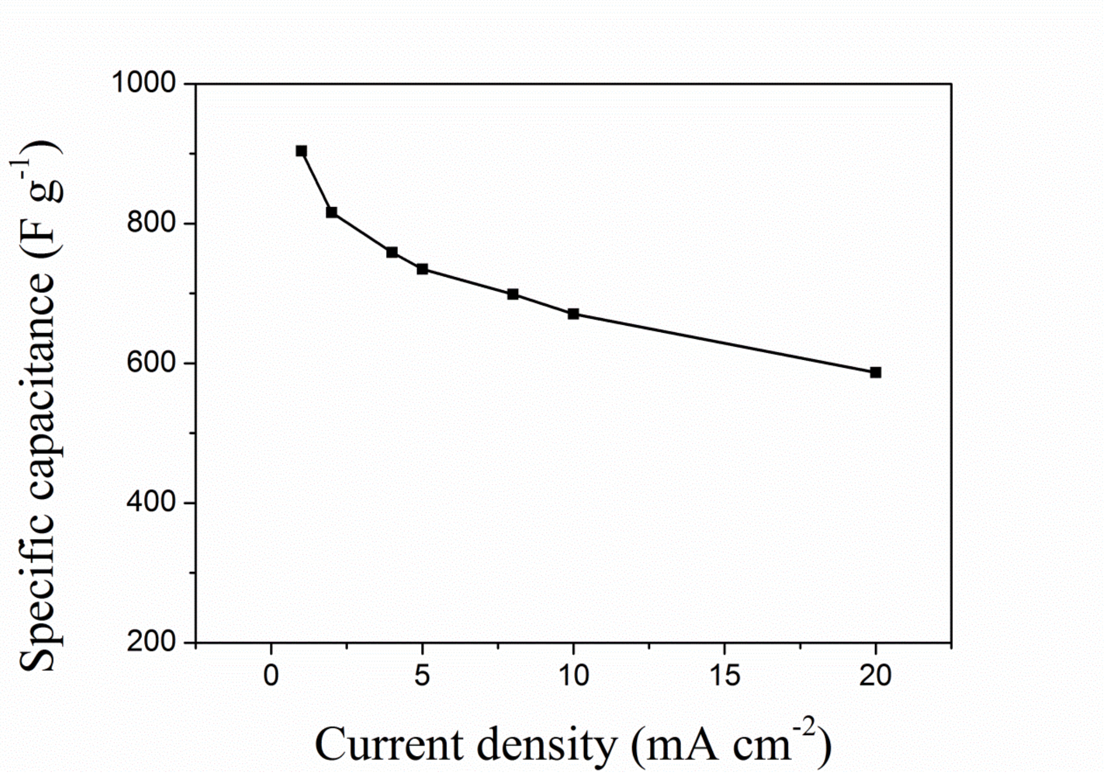

Figure S2. Geometric specific capacitance of core-shell $NiCo_2S_4$@NiO NWAs at different current densities

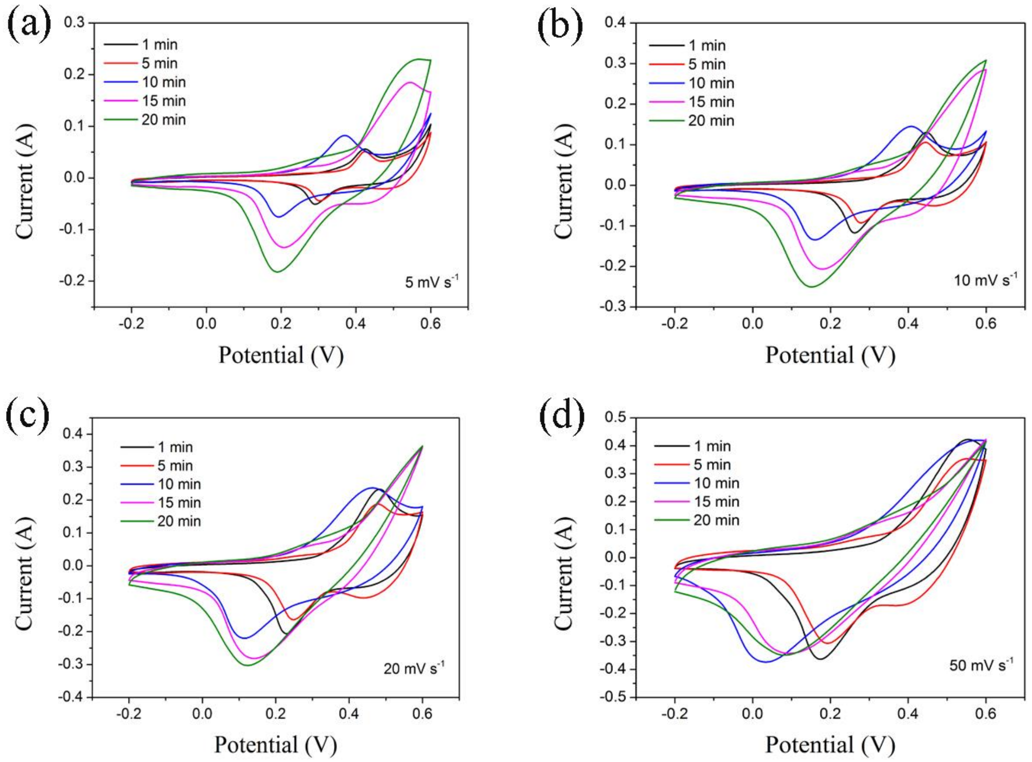

Figure S3. Comparison of CV curves of the $NiCo_2S_4$@NiO NWAs with different electrochemical deposition time and scan rates. (a) Scan rate of 5 mV s^{-1}. (b) Scan rate of 10 mV s^{-1}. (c) Scan rate of 20 mV s^{-1}. (d) Scan rate of 50 mV s^{-1}

$NiCo_2S_4$@NiO electrodes were fabricated with different electrochemical deposition durations, including 1 minute, 5 minutes, 10 minutes, 15 minutes and 20 minutes, respectively. Their cyclic voltammetry properties were analyzed to ensure best electrochemical deposition duration, as shown in Figure. S3. It was observed that the capacitance was increased initially with the increase of deposition time. However, further increasing the deposition time up to 15 minutes, the total capacitance was decreased and the CV curves of the electrodes had deformed even at a low scan rate, which might due to the increase of thickness and density of NiO nanosheets. Too thick and dense NiO nanosheets may limit the contact between the active materials and

electrolyte, prevent the ion penetration to the inner core of $NiCo_2S_4$, decrease the electrode conductivity. Based on the CV curve analysis, we chose 10 minutes of electrochemical deposition as the optimal deposition duration. In the manuscript, all of the $NiCo_2S_4$@NiO NWAs related tests were based on the deposition of 10 minutes.
